# Relationships between Affect Recognition, Empathy, Alexithymia, and Co-Occurring Conditions in Autism

**DOI:** 10.3390/brainsci13081161

**Published:** 2023-08-03

**Authors:** Nandita Raman, Sofronia M. Ringold, Aditya Jayashankar, Christiana D. Butera, Emily Kilroy, Laura Harrison, Sharon A. Cermak, Lisa Aziz-Zadeh

**Affiliations:** 1 Division of Occupational Science and Occupational Therapy, University of Southern California, Los Angeles, CA 90089, USA; ramann@usc.edu (N.R.); sringold@usc.edu (S.M.R.); jayashan@usc.edu (A.J.); cbutera@usc.edu (C.D.B.); emilykilroy@gmail.com (E.K.); lauraloesch09@gmail.com (L.H.); cermak@usc.edu (S.A.C.); 2 Brain and Creativity Institute, Dornsife College of Letters, Arts and Sciences, University of Southern California, Los Angeles, CA 90089, USA; 3 Department of Pediatrics, Keck School of Medicine, University of Southern California, Los Angeles, CA 90033, USA

**Keywords:** autism, alexithymia, empathy, affect recognition, anxiety, sleep, gastrointestinal issues

## Abstract

Prior studies show differences in empathy and affect-recognition ability between those with autism spectrum disorder (ASD) and typically developing (TD) individuals. Autistic individuals also exhibit increased behavioral, gastrointestinal, and sleep issues. In the current study, we explored the differences in empathy and affect recognition between the ASD and TD groups; and we investigated their associations with conditions co-occurring in ASD. A total of 56 TD and 57 ASD children (8–17 years) were included. As compared to the TD group, the ASD group showed lower scores for affect recognition and perspective taking (PT) and higher scores for personal distress (PD). Interestingly, results from hierarchical linear regressions suggested that disparities in the PD and PT between the groups were primarily attributable to attenuated levels of alexithymia, rather than being mediated by the presence of an autism diagnosis. Differences in affect-recognition ability, however, were mediated by both an autism diagnosis and alexithymia. We also found significant correlations between empathy and affect recognition and measures of related conditions common in ASD. Alexithymia, hence, contributes to difficulties in empathy while both alexithymia and autism are associated with affect-recognition ability in ASD. Additionally, the association between affect recognition and empathic ability with co-occurring conditions in ASD needs to be considered during assessments and interventions.

## 1. Introduction

Autism spectrum disorder (ASD) is characterized by deficits in social communication and interaction and the occurrence of restricted and repetitive behaviors [1]. Its prevalence is estimated to affect one in thirty-six children, with males being four times more likely to be diagnosed compared to females [2]. While there is a great range of heterogeneity in symptomatology, autism is commonly associated with reduced socio-emotional reciprocity and a failure to initiate or respond to social interactions. Further, about 40–65% of autistic individuals have alexithymia, compared to a prevalence of 10–18% in the TD population [3,4,5]. Alexithymia refers to a condition characterized by difficulty in identifying and expressing one’s feelings [6]. Other commonly co-occurring conditions in this population include anxiety, gastrointestinal symptoms, and impaired sleep quality. While there are some studies indicating an association between empathy and anxiety [7], and empathy and repetitive behavioral patterns [8], the relationship between aspects of emotion processing (empathy, affect recognition, alexithymia) and these conditions remains poorly understood. To fill this gap, in the current study, we specifically look at how emotion processing is related to other factors in autism.

### 1.1. Empathy

Empathy plays an important role in shaping human social interactions. Cognitive empathy is defined as understanding another person’s perspective using mentalizing processes, such as role taking, while affective empathy is the feeling of emotional resonance with what others are experiencing (feeling what they are feeling [9]). Together, these aspects of empathy help shape our reactions and responses to people and situations, helping us effectively interact with other people.

Individuals with ASD have been reported to have differences in aspects of empathy [9,10,11]. While several studies suggest a decreased cognitive-empathy ability in autistic individuals as compared to TD individuals [12,13,14], there is conflicting data regarding affective empathy. While some studies show that autistic populations may have an increased affective-empathy ability compared to TD populations, particularly with personal distress [12,15,16], some studies find no difference or decreased affective empathy when considering alexithymia difficulties [17,18]. Therefore, it has been suggested that specific components of affective empathy (empathetic concern and personal distress) may need to be examined separately as they may show different patterns in autism [11,16]. Further, studies indicate that differences in empathy may be related to the co-occurring diagnosis of alexithymia rather than the diagnosis of autism. This theory is commonly called the “Alexithymia hypothesis” [16,19].

### 1.2. Facial Affect Recognition

One important component of social interaction and communication is the ability to identify the emotions of other people by their facial expressions (facial affect or emotion recognition). Autistic children may have increased difficulty with facial affect recognition, which could impact their social interaction and communication [20,21]. Behaviorally, autistic individuals display increased attention bias and hypervigilance when viewing negative facial expressions [22] and show difficulties with specific negative emotions (sadness) above and beyond the differences in face processing commonly seen in autism. Neuroimaging studies also support the theory of differential patterns of neural activation in autistic individuals as compared to TD individuals during facial affect recognition [23,24,25]. In a large meta-analysis of 13 neuroimaging studies, results indicated that autistic people showed idiosyncratic neural processing of other people’s facial emotions compared to a TD group [26]. However, some studies indicate that the difference in affect recognition may be attributed to alexithymia, rather than the diagnosis of autism [19,27]. We discuss this further below.

### 1.3. Alexithymia

#### 1.3.1. Alexithymia and Affect Recognition

As mentioned earlier, some researchers have proposed that the presence of alexithymia, instead of autism per se, is associated with the difficulties in affect recognition and empathic processing commonly seen in autism (alexithymia hypothesis) [19]. For example, Bird and Cook [19] found that when controlling for alexithymia, there was no difference between TD and ASD groups in affect-recognition ability. Further, using regression analysis, it was found that alexithymia, and not an autism diagnosis, predicted the accuracy of affect recognition [19]. That study also found that individuals with increased levels of alexithymia had an intact ability to detect physical differences between facial expressions (tested through the task of determining if two stimuli are identical, without labeling it) but had difficulties interpreting the expression. Further, in support of the alexithymia hypothesis for affect recognition, a study investigating dynamic facial affect recognition in autistic females found higher scores of alexithymia to be associated with less-accurate emotion recognition [28]. In addition, another study on autistic individuals suggested that alexithymia was associated with poor affect recognition and that the presence of alexithymia contributed to emotional processing difficulties [29]. Similar findings were found in a TD group, with difficulty with identifying emotions for the self (high alexithymia) identified as significantly related to difficulty with recognizing emotions in others (low affect recognition) [6]. However, Keating et al. [30] found that autistic traits, and not alexithymia, were a significant predictor of accuracy for the recognition of angry facial expressions. The authors suggest that while alexithymia may contribute to a higher intensity of responses to all emotions (correct and incorrect), it does not predict the accuracy of the recognition of emotions. Neuronally, there is evidence that, in autism, alexithymia severity is related to differential neural patterns when looking at facial expressions. In a neuroimaging study, Butera et al. [16] found that when autistic individuals looked at facial expressions, alexithymia severity was significantly associated with reduced interhemispheric neural functional connectivity. Hence, while there is some strong evidence to support the alexithymia hypothesis, there are some conflicting findings as well.

#### 1.3.2. Alexithymia and Empathic Ability

There is also varied evidence for the alexithymia hypothesis for empathic processing. Butera et al. [16] found that alexithymia contributed to personal distress and empathetic concern above an ASD diagnosis but did not look at relationships between alexithymia and cognitive empathy. Mul et al. [14] found that alexithymia contributed to differences in empathy between the TD and ASD groups for affective empathy but not for cognitive empathy. In contrast to both former studies, Shah et al. [31], found that an ASD diagnosis was a stronger predictor of affective and cognitive empathy compared to alexithymia. It is important to note that the study did not examine the components of affective (personal distress and empathic concern) and cognitive (perspective taking and fantasy) empathy separately.

Neuroimaging studies examining alexithymia and empathy have found that differential activation between the TD and ASD groups in some brain regions/networks may be attributed to alexithymia. Bird et al. [32] found alexithymia to be correlated with lower anterior insula activation during empathy regarding pain in both the TD and ASD groups, suggesting that increased levels of alexithymia, and not autism, were predictive of reduced activity in emotional-brain regions. In line with this theory, Lassalle et al. [4] found an inverse correlation between levels of alexithymia and brain activity during empathy for pain tasks in brain regions commonly found to be involved in cognitive and emotional empathy (anterior insula, medial prefrontal cortex, thalamus, and inferior frontal gyrus). Thus, while there was decreased activity in these brain regions in the ASD group as compared to the TD group, no difference was noticed when controlling for alexithymia. Taken together, there is conflicting behavioral data for the alexithymia hypothesis for empathic ability while the neuroimaging data seems to support the hypothesis that affective empathy is related to empathy.

### 1.4. Role of Verbal IQ in Emotion Processing

Prior studies indicate the need for considering verbal and nonverbal IQ separately, in addition to the full-scale IQ (FSIQ) [33]. This may be particularly important for research focusing on alexithymia as it essentially involves language processing in voicing one’s emotional experiences. Indeed, studies examining the relationship between verbal and nonverbal IQ and alexithymia show contrasting results. A study by Montebarocci [34] on TD adults found that individuals with higher alexithymia show significantly lower verbal IQ scores as compared to individuals with lower alexithymia. In contrast, Lane et al. [35] found alexithymia to be associated with affect recognition in both verbal and nonverbal tasks in the TD group. A review by Sivathasan et al. [3] suggests that verbal IQ has a greater influence, compared to alexithymia, on emotion recognition and there is a possible relationship between reduced emotion language processing and increased alexithymia [36]. Verbal IQ, hence, would need to be considered in studies related to affect recognition and alexithymia.

### 1.5. Conditions Co-Occurring with ASD

In ASD, there is a high co-occurrence of anxiety disorder (29.7–49.6% of autistic children) [37] and sleep disturbances (44–83% of autistic children) [38,39]. There is also an increased occurrence of gastrointestinal (GI) symptoms in autism [40], with GI symptoms significantly related to ASD behavioral symptoms [41,42]. Further, ASD behavioral symptoms are significantly related to anxiety and sleep quality [43]. In autism, differences in empathetic processing and affect recognition are known to affect activities of daily living, in general [44]. However, to our knowledge, specific relationships between empathy and affect recognition and factors such as sleep quality, anxiety, and GI disturbances have not been explored.

### 1.6. Current Study

The overarching aim of this study is to better understand the differences in empathy and affect recognition in TD and autistic children and how they are related to ASD symptomology and co-occurring conditions common in ASD. We explore factors such as anxiety, alexithymia, repetitive behaviors, and aspects of daily living, such as gastrointestinal issues, sleep quality, and behavioral issues. In addition, we consider the impact of full-scale IQ, the verbal comprehension index, and the perceptual reasoning index in these potential relationships.

Thus, in this study, we aim to examine the relationships between empathy, affect recognition, and associated factors in autistic children. Building upon our prior research [16,45,46,47], we hypothesize that: (1) autistic children will exhibit reduced scores in cognitive empathy and affect recognition, while displaying increased personal distress (affective empathy), compared to typically developing (TD) individuals; (2) there will be a significant positive correlation between affective empathy and affect recognition, within and across groups, based on the theoretical link between empathic ability and recognizing others’ affective experiences; (3) increased levels of alexithymia will be associated with poorer affect recognition and empathy across groups, aligning with the established connection between alexithymia and impaired emotional processing [19]; and (4) impairments in affect recognition and aspects of empathy in the ASD group will be associated with an increase in ASD symptomologies, such as emotional/behavioral problems, repetitive interests, and co-occurring conditions, including anxiety, gastrointestinal symptoms, and impaired sleep quality. By investigating these hypotheses, we aim to enhance our understanding of the complex interplay between empathy, affect recognition, and associated factors in autistic individuals.

Further, understanding relationships between symptomologies can be used to better inform intervention strategies. For example, in autism, there is increasing research on technology-based interventions designed to improve affect recognition, regulation, and empathic ability. Such technology-based interventions include video modeling (e.g., recording videos of the self or a peer to later watch and perform) and virtual reality techniques (e.g., interactive situation-based learning opportunities). One study found that modeling based on recorded self-recordings was associated with faster acquisition of affect recognition and empathic responses skills compared to peer-video recording modeling [48]. Preliminary studies have found this method was effective in teaching children to recognize and label emotions, even in nonverbal participants [49]. In addition, prior studies indicated that virtual reality was effective in emotion regulation and management and can also lead to the generalization of skills to nonpracticed situations [50,51,52]. Such techniques may be especially effective in children as they may tap into their fascination with exploring technology [53]. Understanding how affect recognition and empathic processing may be related to co-occurring conditions common to autism could help inform intervention strategies.

## 2. Materials and Methods

Data were collected from 110 children between the ages of 8 and 17 years old, for either the typically developing (TD) group (*n* = 54, mean age = 11.39 ± 1.68 years) or the autism spectrum disorder (ASD) group (*n* = 56, mean age = 11.9 ± 2.28 years). The Institutional Review Board at the University of Southern California approved this study. This study was part of a larger study that included neuroimaging and microbiota collection; thus, some of the inclusion and exclusion criteria reflect the criteria for those studies. The inclusion criteria for all participants were: (a) born after 36 weeks of gestation; (b) an IQ of 80 or higher on the full-scale IQ segment of the Wechsler abbreviated scale of intelligence (WASI—2nd edition) or the verbal comprehension index (VCI); (c) fluent in English with at least one parent also fluent in English; and (d) right-handed, as measured by a modified Oldfield questionnaire [54]. The exclusion criteria included: (a) a history of brain injury, concussion, or neural malformations; (b) recent epileptic seizure; (c) previous or current diagnosis of any other major neurological, psychiatric, or developmental disorders; and (d) presence of factors that resulted in MRI incompatibilities, such as having metal braces, metal implants, or claustrophobia.

The TD group included 54 participants (23 females, 31 males) who were screened using the parent-reported social responsiveness scale-2nd edition (SRS-2) and Conners-3. Participants who had a T-score of more than 60 on the SRS (indicating a risk of ASD) and scored more than 65 on the Conners (indicating a risk of attention deficit hyperactive disorder) were excluded from this study. Participants having any major psychological or neurological diagnosis, or a first-degree relative with ASD, were also excluded from this study.

The ASD group included 56 participants (16 females, 40 males). The autism diagnostic observation schedule—2nd edition (ADOS-2) and autism diagnostic interview-revised (ADI-R) were utilized as inclusion criteria.

### 2.1. Behavioral Measures

#### 2.1.1. Inclusion Measures

##### Wechsler Abbreviated Scale of Intelligence—2nd Edition (WASI-II)

The Wechsler abbreviated scale of intelligence (WASI—2nd edition), designed for children and adults between 6 and 89 years old, was used to measure IQ and was a screener for eligibility. All participants had an IQ of 80 or higher. The scores of the vocabulary and similarities subtest were combined to compute the verbal comprehension index (VCI) composite scores; the matrix and block design subtests were combined to compute the perceptual reasoning index (PRI) composite score. All four subtests were reflected in the full-scale IQ-four (FSIQ-4) test [55].

#### 2.1.2. Measures of Autism Severity

##### Autism Diagnostic Observation Schedule—2nd Edition (ADOS-2)

The autism diagnostic observation schedule (ADOS) is a semi-structured, standardized assessment used to measure communication, social interaction, play/imagination, and restricted and repetitive behaviors (ADOS-2) [56]. The ADOS-2 is considered a gold standard assessment measure for ASD [57]. The scale was used as an inclusion criterion for participants in the ASD group and was administered by trained lab personnel. Higher scores on the ADOS-2 suggest increased autism severity.

##### Autism Diagnostic Interview-Revised (ADI-R)

The ADIR-R is a standardized, semi-structured interview for parents and caregivers of children and adults who have a possible diagnosis of autism [58]. The measure has separate scores for the areas of communication; social skills; and restricted, repetitive, and stereotyped behavior. The scale was administered by trained lab personnel. Higher scores on the ADI-R indicate increased autism severity.

##### The Social Responsiveness Scale (SRS-2)

The SRS-2 is a parent/teacher-report scale that is used as a screener and an adjunct in clinical diagnosis for autistic individuals (SRS-2) [59]. The scale was used as a screener to assess social impairment. The scale consists of 65 items rated on a 4-point Likert scale. A T-score of less than 59 is normal, T-scores between 60 and 75 suggest mild/moderate social impairment, and T-scores of 76 and above indicate severe social impairment [60].

##### Conners Parent Rating Scale (Conners-3)

The Conners-3 (CPRS) [61] is a parent-reported scale used as a screener for symptoms of attention deficit hyperactivity disorder (ADHD): hyperactivity, impulsivity, and inattention. The scale contains 10 items rated on a 4-point scale based on how true the statements are for their child over the past month. Higher scores on the Conners-3 indicate an increased probability of behavioral and emotional problems and a risk of ADHD. TD participants with T-scores of 65 or higher were excluded from this study.

#### 2.1.3. Measures of Empathy

##### Interpersonal Reactivity Index (IRI)

The interpersonal reactivity index (IRI) was used to assess empathy. The scale is a 28-item, self-report measure consisting of 4 7-item subscales: perspective taking, personal distress, empathetic concern, and fantasy. The perspective-taking and fantasy subscales measure cognitive empathy while the personal-distress and empathetic-concern subscales measure affective empathy [62]. Items are rated on a 5-point scale based on the extent to which the individual believes the statement describes them. Higher scores on the IRI indicate higher empathic skills.

#### 2.1.4. Measures of Affect Recognition

##### NEPSY-II

The neurophysiological assessment—second edition (NEPSY-II) [63] was used to assess facial affect recognition. It includes the task of asking the child to recognize similar affects from the photographs of children’s faces with different affects, which assesses affect identification, and the task of viewing a photograph briefly and recognizing photographs of similar affects, which assesses recognition memory for affect. The scores of both tasks are combined and scaled (age-normed). Higher scores on the NEPSY-II indicate a better affect-recognition ability.

#### 2.1.5. Measure of Alexithymia

##### The Alexithymia Questionnaire for Children

The alexithymia questionnaire for children was developed from the Toronto alexithymia scale (TAS-20) [64]. It comprises 20 short statements about how a person may think about the way they feel. The participant is asked to rate how well the statement fits them on a 3-point scale, where 1 indicates ‘Not true’, 2 indicates ‘Sometimes true’, and 3 indicates ‘Often true’. The scale has three subtests: (1) difficulty identifying feelings, (2) difficulty communicating feelings, and (3) externally oriented thinking [64]. Due to the low Cronbach’s alpha for externally oriented thinking, we only used the difficulty identifying feelings and difficulty communicating feelings sub scores and the total of these two factors for this study [65]. Higher scores indicate greater alexithymia.

#### 2.1.6. Measures of ASD Symptomologies and Related Conditions

##### Repetitive Behavior Scale (RBS)—Revised (RBS-R)

The RBS-R is a measure developed to assess repetitive behavior patterns in ASD. The scale measures behaviors using 6 factors: stereotyped behavior, self-injurious behavior, compulsive behavior, ritualistic behavior, sameness behavior, and restricted behavior. Each factor contains a list of behaviors and the participant is asked to rate them on a 4-point scale based on the presence, frequency, and degree of impairment due to the behavior. A score of 0 indicates ‘behavior does not occur’, 1 indicates ‘behavior occurs and is a mild problem’, 2 indicates ‘behavior occurs and is a moderate problem’, and 3 indicates ‘behavior occurs and is a severe problem’ [66]. Higher scores indicate more repetitive behaviors.

##### Child Behavior Checklist (CBCL)

The CBCL is a parent-report measure used to identify the presence of behavioral and emotional problems in children ages 6–18. The scale measures competence in four domains (activities, social, school, and total competence) and higher-order factors (internalizing and externalizing) [67]. The scale consists of 113 questions, which are scored on a 3-point Likert scale: 0 indicates ‘absent’, 1 implies ‘occurs sometimes’, and 2 implies ‘occurs often’. Higher scores indicate an increased occurrence of the identified problems.

##### Screen for Child Anxiety Related Emotional Disorders (SCARED)

The SCARED is a self- and parent-report instrument that was used to screen for childhood anxiety disorders. The instrument contains 38 items rated on a 3-point scale where 0 indicates ‘not true or hardly ever true’, 1 indicates ‘sometimes true’, and 2 indicates ‘true or often true’ [68]. Higher scores indicate more anxiety.

##### Gastrointestinal Symptom Rating Scale (GSRS)

The GSRS is a disease-specific self-report instrument used to assess the presence of gastrointestinal symptoms [69]. The scale contains 15 questions answered on a 7-point scale indicating the level of discomfort where 1 implies no discomfort and 7 implies very severe discomfort. A higher score indicates greater severity of the symptoms [70].

##### Adolescent Sleep–Wake Scale (ASWS)

The ASWS—short form, a self-report form for children between the ages of 12 and 18 years, was used to assess sleep quality [71]. The scale consists of 10 items, rated on a 6-point scale based on their frequency over the past month. The total score was used for the analyses of this study. Higher scores indicate better sleep quality.

### 2.2. Data Analysis

Study data were collected and managed using REDCap electronic data capture tools hosted at the University of Southern California [72,73]. Data were analyzed using IBM SPSS Statistics (Version 28). Baseline differences in age and IQ were analyzed using the independent sample t-test and sex differences were analyzed using the Pearson chi-square test. The differences in empathy, affect recognition, and functional measures between the TD and ASD groups were tested while controlling for age, sex, and IQ, using the ANCOVA. Multiple comparison correction was performed using the Bonferroni method when comparing the estimated marginal means for each group [74]. Pearson’s correlation was used to measure relationships between empathy, age, and IQ. Pearson’s partial correlation was used to understand how empathy and affect recognition may be related to ASD symptomologies and co-occurring conditions, such as anxiety, gastrointestinal disturbances, and sleep quality while controlling for age, sex, and IQ. Multiple comparison correction was performed using the Bonferroni method, determining the significance of the corrected alpha values [74]. Hierarchical linear regression was performed to determine factors contributing to variations in empathy and affect recognition.

## 3. Results

### 3.1. Between-Group Differences

#### 3.1.1. Demographics

There were no differences seen in age and sex between the typically developing (TD) and autism spectrum disorder (ASD) groups (t = −1.348, *p* = 0.181, and 𝝌 = 2.362, *p* = 0.124, respectively). The groups differed significantly in full-scale IQ (FSIQ-4), with higher scores in the TD group (t = 4.291, *p* < 0.001), see Table 1. Therefore, we controlled for FSIQ-4, in all our analyses. The verbal comprehension intelligence index (VCI) and perceptual reasoning index (PRI) showed a similar pattern of significantly higher scores in the TD group as compared to the ASD group (t = 4.462, *p* < 0.001 and t = 2.890, *p* = 0.002, respectively). Hence, for assessments where verbal IQ may impact responses (affect recognition), we also looked at the impact of VCI and PRI separately.

#### 3.1.2. Empathy

The ASD group scored significantly higher in personal distress than the TD group (see Table 2), controlling for age, sex, and FSIQ-4 (*p* = 0.027). However, the between-group difference was no longer significant when additionally controlling for alexithymia (*p* = 0.379).

The TD group scored significantly higher on perspective taking than the ASD group, controlling for age, sex, and FSIQ-4 (*p* = 0.034). However, the between-group differences did not remain significant when additionally controlling for alexithymia (*p* = 0.148). No other between-group significant differences in empathy were found.

#### 3.1.3. Affect Recognition 

There is a significant difference in the affect recognition scaled scores between the TD and ASD groups (*p* = 0.001) when controlling for age, sex, and FSIQ-4, with higher scores in the TD group as compared to the ASD group (see Table 2). Interestingly, the difference also remains significant when additionally controlling for alexithymia (*p* = 0.015) and when selectively controlling for PRI and VCI (in addition to age, sex, and alexithymia; *p* < 0.001, in both).

#### 3.1.4. Theory of Mind (ToM)

The TD group scored significantly higher than the ASD group on ToM ability when controlling for age, sex, and FSIQ-4 (*p* < 0.001) and when additionally controlling for alexithymia (*p* < 0.001; see Table 2).

#### 3.1.5. Alexithymia

The ASD group had significantly higher the alexithymia two-factor scores when controlling for age, sex, and FSIQ (*p* = 0.002). Looking more closely, as the communication subscore was significant but the identification subscale was not, it appeared the result of higher alexithymia in the ASD group was driven by the communication subscale; but, further analysis is needed to confirm this.

#### 3.1.6. Other Behavioral Measures

Significant group differences were found for RBS and CBCL in the expected directions (see Table 3). Regarding the gastrointestinal symptom rating scale (GSRS), significantly more GI issues in the ASD group than in the TD group were observed when controlling for age, sex, and FSIQ-4 (*p* = 0.012). There was a trend toward significance on the adolescent sleep–wake scale (ASWS) when controlling for age, sex, and FSIQ-4 (*p* = 0.061), with the TD group having better sleep quality than the ASD group. No other significant relationships or trends were found when controlling for age, sex, and FSIQ.

### 3.2. Correlations of Behavioral Measures

#### 3.2.1. Correlations with Affect Recognition in ASD Group

##### Correlation of Affect Recognition with Demographics and Alexithymia

As Supplementary Table S1 shows, there was a significant positive correlation between FSIQ-4 and affect recognition in the ASD group (r = 0.377, *p* = 0.004). For subtests, a marginally significantly positive correlation was found between affect recognition and VCI in the TD group (r = 0.299, *p* = 0.028). For affect recognition and PRI, a significant positive correlation was found only in the ASD group (r = 0.354, *p* = 0.007).

As shown in Table 4, in the ASD group, there was a significant negative correlation between affect recognition and alexithymia (identification) when controlling for age and sex (r = −0.312, *p* = 0.022); but, the correlation did not remain significant when additionally controlling for FSIQ-4 (r = −0.195, *p* = 0.162). Similar patterns were seen when controlling for PRI and VCI instead of the FSIQ-4. For TD group correlations, see Supplementary Table S2.

##### Correlation of Affect Recognition with Empathy

No significant correlations or trends between the empathy scales and affect recognition were found when controlling for age, sex, and FSIQ-4 in the ASD (Table 4) or TD groups (see Supplementary Table S2).

##### Correlation of Affect Recognition with Other Measures

As Supplementary Table S3b shows, in the ASD group, there was a significant positive correlation between affect recognition and the ASWS scores when controlling for age, sex, and IQ (r = 0.474, *p* = 0.012). No other significant correlations were found. There were no significant correlations seen in the TD group (see Supplementary Table S3a,b).

#### 3.2.2. Correlations with Empathy in the ASD Group

##### Correlations between Affective and Cognitive Empathy

In the ASD group, there was a significant positive correlation seen between empathetic concern (affective empathy) and fantasy (cognitive empathy), when controlling for age, sex, and FSIQ (r = 0.543, *p* < 0.001), and between empathetic concern (affective empathy) and perspective taking (cognitive empathy), when controlling for age, sex and FSIQ (r = 0.320, *p* = 0.020). Similar correlations were seen in the TD group as well.

##### Personal Distress

**Figure 1:**
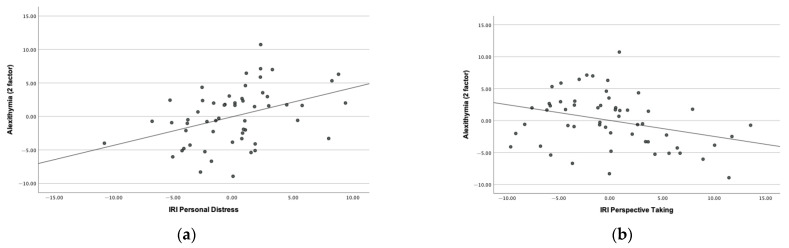
(**a**) Partial correlations of alexithymia and personal distress in the ASD group when controlling for age, sex, and FSIQ (r = 0.401, *p* = 0.003); (**b**) Partial correlations of alexithymia and perspective taking in the ASD group when controlling for age, sex and FSIQ (r = –0.321, *p* = 0.019).

There was no significant correlation between personal distress and age or IQ in the TD or ASD groups (see Supplementary Table S4). There was a statistically significant positive correlation between personal distress and alexithymia in the ASD group when controlling for age, sex, and FSIQ-4 (r = 0.401, *p* = 0.003) (see Figure 1a). This effect was common to both the alexithymia-identification and alexithymia-communication subscales (r = 0.380, *p* = 0.005 and r = 0.309, *p* = 0.024, respectively) (see Supplementary Table S12). There was a positive correlation between personal distress and child-reported anxiety (r = 0.581, *p* ≤ 0.001), as well as a significant negative correlation with sleep quality (r = −0.479, *p* = 0.011), when controlling for age, sex, and FSIQ-4 in the ASD group (see Supplementary Table S5b). Similar correlations between personal distress and alexithymia were seen in the TD group, (see Supplementary Table S5b).

##### Empathetic Concern

There was no significant correlation between personal distress and age or IQ in the TD or ASD groups (see Supplementary Table S6). Additionally, there was a marginally significant negative correlation between alexithymia (communication) and empathetic concern in the ASD group when controlling for age, sex, and IQ (r = −0.523, *p* = <0.001) (see Supplementary Table S12). There were no other significant correlations between empathetic concern and other measures (see Supplementary Table S7a,b).

##### Perspective Taking

There was a significant positive correlation seen between age and perspective taking in the ASD group (r = 0.326, *p* = 0.014) but not in the TD group (r = 0.215, *p* = 0.118) (see Supplementary Table S8). In the ASD group, there was a significant negative correlation seen between alexithymia (communication and 2 factor) and perspective taking when controlling for age, sex, and IQ (r = −0.340, *p* = 0.013; and r = −0.321, *p* = 0.019) (see Supplementary Table S12, see Figure 1b). There was a trend for a negative correlation seen between repetitive patterns of ritualistic behavior (r = −0.322, *p* = 0.021) and perspective taking when controlling for age, sex, and FSIQ (see Supplementary Table S9a,b).

##### Fantasy

There was no significant correlation between fantasy and age or IQ in the TD and ASD groups (see Supplementary Table S10). There was a significant negative correlation between alexithymia (communication) and fantasy in the ASD group when controlling for age, sex, and FSIQ (r = −0.346, *p* = 0.011) (see Supplementary Table S12). There were no correlations seen with other measures in the ASD group (Supplementary Table S11a,b). For TD correlations, see Supplementary Table S11a,b.

### 3.3. Hierarchical Regression Analysis

A hierarchical linear regression was run across all participants to see if the variation in affect recognition, personal distress, and empathy between the TD and ASD groups was explained by variations in alexithymia.

#### 3.3.1. Affect Recognition

Model 1, which included age, sex, FSIQ, and alexithymia (2 factor), was significant and explained 30.1% of the variance in affect recognition across participants (see Table 5). The addition of group membership to this model explained an additional 3.9% of the variation; group membership was a significant individual predictor (*p* = 0.015), with the TD group having an increased ability as compared to the ASD group. An increase in alexithymia was associated with decreased affect-recognition ability (*p* = 0.016). The results suggest that autism diagnoses contribute significantly to differences in affect recognition between groups, as do alexithymia severity and IQ.

#### 3.3.2. Empathy

##### Personal Distress

Model 1, which includes age, sex, FSIQ, and alexithymia (2 factor), was significant and explained 30.1% of the variance in personal distress across participants (see Table 6). The addition of affect-recognition ability to this model did not contribute to the model (*p* = 0.857). Further, adding group membership to this model explained an additional 0.5% of the variation but was not found to be an individual predictor as well (*p* = 0.391). An increase in alexithymia was associated with increased personal distress (*p* < 0.001). The results suggest that autism diagnoses and affect recognition do not contribute to differences in personal distress between the groups, beyond alexithymia severity and sex.

##### Perspective Taking

Model 1, which includes age, sex, FSIQ, and alexithymia (2 factor), explained 17.9% of the variance in perspective taking across participants (see Table 7). The addition of a group to this model explained an additional 1.6% of the variation in perspective taking; but, an autism diagnosis was not an individual predictor (*p* = 0.148). The results suggest that an autism diagnosis does not contribute to differences in perspective taking between groups, beyond alexithymia severity, and age.

## 4. Discussion

This study aimed to understand potential differences in affect recognition and empathy in autistic children and how those differences may be impacted by alexithymia and other co-occurring conditions in autism. Our results indicate that compared to the TD group, the ASD group scores significantly lower in affect recognition and perspective-taking ability but significantly higher in the personal distress component of affective empathy. The results indicate that aspects of cognitive- and affective-empathy ability are influenced by alexithymia severity. In contrast, affect-recognition ability appears to be influenced by both group membership and alexithymia severity. These results are discussed in more depth below.

### 4.1. Affect Recognition 

Our data support prior studies indicating that the ASD group has more difficulty with affect recognition than the TD group [20,21,75]. Importantly, we find that between-group differences in affect recognition remain significant, even after controlling for alexithymia. We found no relationship between alexithymia and affect recognition in the ASD group; although, we did find a significant negative relationship in the TD group. The latter is in line with a prior study involving TD adults, which also indicated that as alexithymia increases, affect-recognition ability decreases [35]. The lack of a significant findings in the ASD group indicates that other factors, beyond alexithymia, are impacting the ability to recognize affect in autism. Accordingly, we found that ASD diagnoses, alexithymia, and IQ contribute to affect-recognition ability. This is in contradiction to the alexithymia hypothesis [19], which predicts that alexithymia severity drives affection-recognition ability, rather than group membership.

When considering the association of affect-recognition ability with other common conditions in ASD symptoms, we found a significant positive correlation with sleep quality. As disturbances in sleep efficiency [76,77] and sleep difficulties [43,78] are common in ASD, future interventions may need to consider emotional-processing issues when considering interventions surrounding the improvement of sleep quality.

In contradiction to our hypotheses, we did not find a relationship between affect recognition and personal distress, perspective taking, and other empathy measures in the TD or ASD groups. This suggests that impairment in aspects of affective empathy, such as personal distress, may not be associated with difficulties in identifying the affective states of others in this group.

Taken together, these results indicate that autistic children have difficulties with nonverbal affect recognition; these difficulties are not restricted to alexithymia severity, but also depend on group membership and IQ and are related to sleep quality. Future studies using other types of affect-recognition tasks (verbal, dynamic stimuli, emotion labeling) are necessary to test the generalizability of the current findings.

### 4.2. Empathy

Personal Distress: In line with previous findings, the ASD group had significantly higher scores on personal distress as compared to the TD group; however, this finding did not remain significant when controlling for alexithymia [16]. We found the model consisting of age, sex, IQ, and alexithymia to explain 30.1% of the variance in personal distress across participants while the addition of group membership (ASD, TD) did not contribute significantly to the difference (only explains an additional 0.5% of the variance). This replicates a prior finding by our group but with double the sample size, as previously reported in Butera et al. [16]. Thus, our findings further support the alexithymia hypothesis with respect to affective empathy, which posits that alexithymia, and not group membership, explains group differences in affective empathy [19].

We next considered the relationship between personal distress and other conditions common in ASD. In line with previous findings, we found autistic children to display higher anxiety than those in the TD group [16]. Further, we found a positive correlation between personal distress and anxiety, which is in line with previous findings on children with ASD [7], indicating that clinicians working with children with high anxiety may also need to be attuned to potentially increased feelings of personal distress in those populations.

Interestingly, we found a significant negative correlation between personal distress and sleep quality in the ASD group. To our knowledge, this is the first report of such an association in ASD; although, previous studies in TD preschoolers [79] and adults [80] have found a significant association between sleep disturbances and quality and empathic ability. As previous research suggested that decreased sleep quality in children may be due to increased anxiety [81], we ran a post hoc mediation analysis to see if anxiety plays an indirect role in the relationship between personal distress and sleep quality; but it was not found to be significant (*p* = 0.136). However, given the correlation between personal distress, anxiety, and sleep quality in autistic children, it may be important to consider these factors together when designing intervention plans for autistic children.

Perspective Taking: Our findings support prior results indicating lower scores in cognitive empathy in the ASD group as compared to the TD group [12,13,14,16]. Interestingly, we show that this difference does not remain when controlling for alexithymia; the hierarchical regression analysis indicates that significant differences in perspective taking between the TD and ASD groups may be more closely related to alexithymia than group membership. Thus, our results support the alexithymia hypothesis for perspective taking in addition to personal distress, which, to our knowledge, is a novel finding.

Prior studies that considered the role of alexithymia [31,82] were conducted in adults and mostly utilized an autism group defined by autistic traits, rather than a clinical diagnosis confirmed by ADOS and ADI-R, which was the criteria used in the current study. Thus, this is the first study in children diagnosed with autism that shows perspective-taking to be more related to alexithymia than the presence of autism. Although future studies in large and more-diverse samples are necessary, the results are intriguing as they indicate that a common primary symptom—perspective taking—may not be attributed to autism per se, but instead to co-occurring alexithymia.

Within the ASD group, there was a positive correlation between age and perspective taking and this correlation remained significant while controlling for alexithymia, which suggests that cognitive-empathy ability improves as autistic children grow older. Such a result may be due to executive-functioning ability, which greatly improves with age. Further studies are needed to understand better if this is due to neural maturation, social learning, cognitive development, or from therapy.

Finally, we related perspective taking to other co-occurring conditions. We found a trend in which increased perspective-taking ability correlated with decreased repetitive behaviors (ritualistic domains) among autistic children. A previous study by Brett et al. [8] with 301 adults looked at the impact of autistic traits on empathy using the serial mediation model. The results indicated that, while restricted repetitive behavior showed no effect on empathy (affective, cognitive, and personal distress), it may indirectly influence empathy through alexithymia and anxiety. Further research is needed to better understand if repetitive behaviors serve, in part, as an externalization of difficulty with perceiving social situations or if they both stem from other related factors. Nevertheless, it may be useful for therapists to couple interventions focused on repetitive behaviors with perspective-taking skills; although further work is needed.

Empathy Summary: The current study, in part, expands prior work on empathy and alexithymia via our research team [16]. By including a sample size consisting of twice the number of autistic children compared to our previous study, we verify our prior published results indicating that autistic children show significantly increased levels of personal distress and significantly lower levels of perspective taking as compared to typically developing children and that alexithymia severity predicts individual differences in personal distress. A novel finding, to our knowledge, not previously described, is that alexithymia severity also predicts perspective-taking ability across groups, beyond autism diagnoses.

It is possible that social skills, which are affected by autism, are more of a predictor than alexithymia and that alexithymia serves as a proxy for autism severity. Indeed, a study by Shah et al. [31] on TD adults found that although alexithymia partially contributes to empathy (affective and cognitive combined measured using the questionnaire for cognitive and affective empathy), autistic traits measured using the autism quotient scale are more predictive of empathic ability than alexithymia. To test this hypothesis, we ran a post hoc analysis of total empathy scores (combined IRI cognitive- and affective-empathy scores) and autistic traits using the SRS Total T-scores to replicate the analysis of this study. The results show that while the model consisting of age, sex, IQ, and alexithymia was not significant for overall and combined affective domains, the addition of SRS to the model was not significant either. For the personal-distress component of affective empathy in particular, the model consisting of age, sex, IQ, and alexithymia was significant and explained 30.1% of the variance. The model with the addition of SRS Total T-scores, though significant, explained only an addition of 0.1%; the SRS was not found to be a significant individual predictor. We found a similar pattern for empathic concern. For empathetic concern, the model consisting of age, sex, IQ, and alexithymia was not significant (*p* = 0.072) and explained 7.8% of the variance. The model with the addition of SRS Total T-scores was not significant (*p* = 0.128) and did not explain any additional variance; SRS was not found to be a significant individual predictor (*p* = 0.904). With respect to overall cognitive empathy, while the model consisting of age, sex, IQ, and alexithymia was significant and explained 11.1% of the variance, the addition of SRS to the model, though significant, explained only an additional 0.7% of the variance. Focusing on perspective taking, the age, sex, IQ, and alexithymia model explained 17.9% of the variance and the addition of SRS explained an additional 2%. Hence, the SRS, though important with perspective taking, does not play a greater role than alexithymia in predicting perspective-taking ability. Taken together, our results indicate that autism severity, as measured by the SRS, does not predict scores on empathic ability.

While several studies now have supported the alexithymia hypothesis for aspects of affective empathy, to our knowledge, the finding that aspects of perspective taking are predicted by alexithymia, rather than autism diagnoses or severity, is a novel finding. It indicates that we can no longer think of key aspects of empathic processing—perspective taking or personal distress—as varying with autism, per se, but instead may be more related to the presence of alexithymia. Our findings also support the notion that cognitive and affective empathy vary differently and a combined score should not be used to estimate this measure [16].

### 4.3. Limitations

The current study only includes participants with an IQ of 80 and above on the WASI-II. While we did not find IQ to be a predictor of any of the empathy measures, it would be important to assess these questions in individuals with lower cognitive abilities to expand the generalizability of our conclusions. The second limitation is using self-report measures for alexithymia, empathic ability, sleep quality, and gastrointestinal disturbances. The validity of self-report measures in autism, given the possibility of impaired metacognitive and self-referential abilities, has been previously debated [83,84]. Alternate behavioral measures, such as the alexithymia observation scale for children [85], would help confirm the findings. Lastly, in this study, we used the NEPSY to evaluate affect-recognition ability, which involves a task involving the picture matching of affective facial expressions. Prior studies indicate a difference in results when using verbal and nonverbal affect-recognition assessments. Although we control for verbal IQ in our study, further studies utilizing different affect-recognition tasks would be helpful to understanding the results more fully.

## 5. Conclusions

To our knowledge, this is the first study to show that perspective-taking ability is better predicted by the presence of alexithymia than an autism diagnosis. This result, combined with prior studies supporting the alexithymia hypothesis for affective empathy, indicates that cognitive and emotional empathy may rely on alexithymia, rather than the presence of autism.

By contrast, we found that affect recognition depends on both alexithymia and an autism diagnosis, which indicates that impairments in affect recognition may be a primary feature of autism. Further, we find that personal distress and affect recognition are related to sleep quality and that perspective taking is related to repetitive behavior and anxiety. Thus, future studies may think of clustering symptomologies when thinking about intervention strategies.

## Figures and Tables

**Table 1:** Demographics.

	TD (n = 54) Mean (±SD)	ASD (n = 56) Mean (±SD)	Test Statistics	*p*
Age	11.39 (±1.68)	11.90 (±2.28)	−1.348	0.181
Sex	Females = 23 Males = 31	Females = 16 Males = 40	2.362	0.124
FSIQ-4	118.13 (±13.87)	105.63 (±16.52)	4.291 *	<0.001
PRI	114.37 (±17.44)	105.13 (±16.11)	2.890 *	0.005
VCI	118.83 (±13.52)	105.00 (±18.51)	4.462 *	<0.001

An independent sample t-test was used to analyze between-group differences for demographics. Pearson’s chi-square test was used to analyze differences in sex between the groups. The test statistics found to be significant at a 95% confidence level are marked using *. TD = typically developing, ASD = autism spectrum disorder, FSIQ-4 = full-scale IQ, PRI = perceptual reasoning index, VCI = verbal comprehension index.

**Table 2:** Group differences in empathy, alexithymia, affect recognition, and theory of mind.

	TD	ASD	Controlling for Age, Sex and FSIQ-4		Controlling for Age, Sex, FSIQ-4 and Alexithymia	
	Mean (±SD)	Mean (±SD)	*F*	*p*	*F*	*p*
IRI personal distress TD, n = 54; ASD, n = 56	12.65 (±5.27)	14.41 (±4.33)	5.020 *	0.027	0.781	0.379
IRI empathetic concern TD, n = 54; ASD, n = 56	18.37 (±4.84)	17.16 (±5.52)	0.396	0.530	0.002	0.967
IRI perspective taking TD, n = 54; ASD, n = 56	14.80 (±5.27)	12.59 (±6.01)	4.616 *	0.034	2.126	0.148
IRI fantasy TD, n = 54; ASD, n = 56	17.44 (±5.44)	16.73 (±5.72)	0.010	0.921	0.004	0.948
Alexithymia—Identification TD, n = 54; ASD, n = 56	0.48 (±0.40)	0.66 (±0.40)	3.058	0.083	-	-
Alexithymia—Communication TD, n = 54; ASD, n = 56	0.67 (±0.50)	1 (±0.45)	16.539 *	<0.001	-	-
Alexithymia—2 factor score TD, n = 54; ASD, n = 56	6.72 (±4.80)	9.59 (±4.32)	10.064 *	0.002	-	-
Affect recognition TD, n = 54; ASD, n = 56	11.80 (±2.34)	9.57 (±2.20)	10.767 *	0.001	6.163 *	0.015
Theory of Mind (Total) TD, n = 54; ASD, n = 54	25.39 (±2.00)	4.96 (±0.97)	4080.49 *	<0.001	3716.17 *	<0.001

ANCOVA was used to analyze differences between the TD and ASD groups. The test statistics found to be significant at a 95% confidence level are marked using *. TD = typically developing, ASD = autism spectrum disorder, FSIQ-4 = full-scale IQ, IRI = interpersonal reactivity index.

**Table 3:** Group differences in other measures.

	TD (n = 54)	ASD (n = 56)	Controlling for Age, Sex, and FSIQ-4	
	Mean (±SD)	Mean (±SD)	*F*	*p*
RBS Stereotype subscore TD, n = 53; ASD, n = 56	0.14 (±0.53)	3.59 (±3.08)	44.806 *	<0.001
RBS Self-injury subscore TD, n = 52; ASD, n = 56	0.47 (±1.77)	2.67 (±3.85)	9.078 *	0.003
RBS Compulsive subscore TD, n = 53; ASD, n = 55	0.55 (±1.24)	3.52 (±4.16)	17.154 *	<0.001
RBS Ritual subscore TD, n = 53; ASD, n = 56	0.33 (±0.86)	4.87 (±3.57)	61.335 *	<0.001
RBS Sameness subscore TD, n = 53; ASD, n = 55	0.33 (±0.89)	7.43 (±5.47)	68.618 *	<0.001
RBS Restricted subscore TD, n = 53; ASD, n = 56	0.10 (±0.36)	2.72 (±2.18)	52.573 *	<0.001
SCARED (Parent) Total score TD, n = 53; ASD, n = 54	5.80 (±5.80)	21.33 (±15.48)	38.914 *	<0.001
SCARED (Child) Total score TD, n = 49; ASD, n = 54	20.09 (±13.07)	26.61 (±14.21)	3.131	0.080
CBCL Competence Activities T-score TD, n = 53; ASD, n = 54	49.69 (±9.90)	41.04 (±9.18)	17.081 *	<0.001
CBCL Competence Social T-score TD, n = 53; ASD, n = 53	49.33 (±8.53)	37.16 (±8.93)	30.566 *	<0.001
CBCL Total score TD, n = 53; ASD, n = 53	52.82 (±3.61)	40.51 (±8.05)	64.435 *	<0.001
CBCL Internalizing problems TD, n = 49; ASD, n = 51	50.98 (±9.67)	35.39 (±7.56)	59.667 *	<0.001
CBCL Externalizing problems TD, n = 49; ASD, n = 52	44.84 (±7.89)	62.27 (±10.66)	70.040 *	<0.001
ASWS Total score TD, n = 27; ASD, n = 30	40.29 (±6.25)	53.16 (±10.49)	53.018 *	<0.001
GSRS Total score TD, n = 41; ASD, n = 43	4.39 (±0.71)	4.02 (±0.76)	3.656	0.061

ANCOVA was used to analyze differences between the TD and ASD groups. The test statistics found to be significant at a 95% confidence level are marked using *. TD = typically developing, ASD = autism spectrum disorder, FSIQ-4 = full-scale IQ, RBS = repetitive behavior scale, CBCL = child behavior checklist, ASWS = adolescent sleep–wake scale total score, GSRS = gastrointestinal symptom rating scale total score.

**Table 4:** Correlations between affect recognition and empathy and affect recognition and alexithymia in ASD.

	Not Controlled		Controlled for Age and Sex		Controlled for Age, Sex, and FSIQ-4		Controlled for Age, Sex, and PRI		Controlled for Age, Sex, and VCI		Controlled for Age, Sex, Alexithymia, and FSIQ-4	
	*F*	*p*	*F*	*p*	*F*	*p*	*F*	*p*	*F*	*p*	*F*	*p*
Alexithymia—Identification TD, n = 54; ASD, n = 56	−0.317 *	0.017	−0.312 *	0.022	−0.195	0.162	−0.205	0.142	−0.250	0.071		
Alexithymia—Communication TD, n = 54; ASD, n = 56	−0.066	0.631	−0.054	0.697	−0.094	0.502	−0.014	0.920	−0.136	0.332		
Alexithymia—2 factor score TD, n = 54; ASD, n = 56	−0.241	0.074	−0.233	0.090	−0.171	0.221	−0.138	0.324	−0.228	0.101		
IRI personal distress TD, n = 54; ASD, n = 56	0.138	0.312	0.160	0.248	0.086	0.540	0.064	0.651	0.137	0.329	0.041	0.772
IRI empathetic concern TD, n = 54; ASD, n = 56	−0.264	0.049	−0.215	0.118	−0.148	0.291	−0.169	0.226	−0.160	0.253	−0.088	0.537
IRI perspective taking TD, n = 54; ASD, n = 56	0.149	0.274	0.173	0.211	0.096	0.494	0.126	0.368	0.113	0.422	0.044	0.757
IRI fantasy TD, n = 54; ASD, n = 56	0.103	0.448	0.140	0.314	0.130	0.352	0.129	0.358	0.134	0.339	0.092	0.514

Pearson’s correlation was used to analyze the table, multiple comparison correction was done using Bonferroni’s method. The correlation coefficients which are significant at a 95% confidence level are marked using *. ASD = Autism Spectrum Disorder, FSIQ-4 = Full-scale IQ, PRI = Perceptual Reasoning Index, VCI = Verbal Comprehension Index, IRI = Interpersonal Reactivity Index.

**Table 5:** Hierarchical linear regression for affect recognition.

Variable	Model 1				Model 2			
	*B*	SE *B*	*β*	*p*	*B*	SE *B*	*β*	*p*
Age	−0.150	0.103	−0.119	0.148	−0.123	0.101	−0.099	0.224
Sex	−0.712	0.433	−0.136	0.103	−0.516	0.430	−0.098	0.233
FSIQ	0.062	0.013	0.406	<0.001	0.050	0.013	0.328	<0.001
Alexithymia (2 factor)	−0.143	0.044	−0.271	0.002	−0.110	0.045	−0.208	0.016
Group					−1.145	0.461	−0.228	0.015
Model *p*				<0.001				<0.001
R^2^				0.301				0.340
Change in R^2^								0.039

Hierarchical linear regression for affect recognition was undertaken across groups. Dependent variable: affect recognition; FSIQ-4 = full-scale IQ.

**Table 6:** Hierarchical linear regression for personal distress.

Variable	Model 1				Model 2				Model 3			
	*B*	SE *B*	*β*	*p*	*B*	SE *B*	*β*	*p*	*B*	SE *B*	*β*	*p*
Age	0.091	0.199	0.038	0.647	0.086	0.202	0.036	0.670	0.073	0.202	0.030	0.717
Sex	−2.424	0.837	−0.239	0.005	−2.448	0.852	−0.241	0.005	−2.559	0.863	−0.252	0.004
FSIQ	−0.008	0.024	−0.025	0.759	−0.005	0.027	−0.018	0.844	0.001	0.028	0.002	0.982
Alexithymia (2 factor)	0.472	0.085	0.462	<0.001	0.467	0.089	0.457	<0.001	0.450	0.092	0.440	<0.001
Affect Recognition					−0.034	0.190	−0.018	0.857	0.006	0.195	0.003	0.977
Group									0.815	0.946	0.084	0.391
Model *p*				<0.001				<0.001				<0.001
R^2^				0.301				0.301				0.306
Change in R^2^				<0.001				0.000				0.005

Hierarchical linear regression for personal distress was undertaken across groups. Dependent variable: personal distress; FSIQ-4 = full-scale IQ.

**Table 7:** Hierarchical linear regression for perspective taking.

Variable	Model 1				Model 2			
	*B*	SE *B*	*β*	*p*	*B*	SE *B*	*β*	*p*
Age	0.727	0.253	0.255	0.005	0.766	0.254	0.269	0.003
Sex	1.564	1.069	0.131	0.146	1.853	1.081	0.155	0.089
FSIQ	0.056	0.031	0.161	0.073	0.039	0.033	0.111	0.247
Alexithymia (2 factor)	−0.288	0.108	−0.239	0.009	−0.239	0.113	−0.199	0.036
Group					−1.691	1.160	−0.148	0.148
Model *p*				<0.001				<0.001
R^2^				0.179				0.196
Change in R^2^								0.016

Hierarchical linear regression for affect recognition was undertaken across groups. Dependent variable: affect recognition; FSIQ-4 = full-scale IQ.

## Data Availability

Not applicable.

## Supplementary Materials

The following supporting information can be downloaded at: https://www.mdpi.com/2076-3425/13/8/1161/s1, Table S1: Factors correlated with affect recognition; Table S2: Correlations between affect recognition and empathy and affect recognition and alexithymia in the TD group; Table S3: (a) Correlations between affect recognition and other ASD symptomology in the ASD and TD groups; (b) Correlations between affect recognition and common conditions in ASD, in the TD and ASD groups; Table S4: Correlation of personal distress with age and IQ; Table S5: (a) Correlations between personal distress and other ASD symptomology in the ASD and TD groups; (b) Correlations between personal distress and common conditions in ASD, in the TD and ASD groups; Table S6: Correlation of empathetic concern with age and IQ; Table S7: (a) Correlations between empathetic concern and other ASD symptomology in the ASD and TD groups; (b) Correlations between empathetic concern and common conditions in ASD, in the TD and ASD groups; Table S8: Correlation of perspective taking with age and IQ; Table S9: (a) Correlations between perspective taking and other ASD symptomology in the ASD and TD groups; (b) Correlations between perspective taking and common conditions in ASD, in the TD and ASD groups; Table S10: Correlation of fantasy with age and IQ; Table S11: (a) Correlations between fantasy and other ASD symptomology in the ASD and TD groups; (b) Correlations between fantasy and common conditions in ASD, in the TD and ASD groups; Table S12: (a) Correlations between alexithymia and empathy in the TD group; (b) Correlations between alexithymia and empathy in the ASD group [86,87,88,89,90,91,92,93].
