# Correction: Raman et al. Relationships between Affect Recognition, Empathy, Alexithymia, and Co-Occurring Conditions in Autism. *Brain Sci.* 2023, *13*, 1161

**DOI:** 10.3390/brainsci14070679

**Published:** 2024-07-03

**Authors:** Nandita Raman, Sofronia M. Ringold, Aditya Jayashankar, Christiana D. Butera, Emily Kilroy, Laura Harrison, Sharon A. Cermak, Lisa Aziz-Zadeh

**Affiliations:** 1 Division of Occupational Science and Occupational Therapy, University of Southern California, Los Angeles, CA 90089, USA; ramann@usc.edu (N.R.); sringold@usc.edu (S.M.R.); jayashan@usc.edu (A.J.); cbutera@usc.edu (C.D.B.); emilykilroy@gmail.com (E.K.); lauraloesch09@gmail.com (L.H.); cermak@usc.edu (S.A.C.); 2 Brain and Creativity Institute, Dornsife College of Letters, Arts and Sciences, University of Southern California, Los Angeles, CA 90089, USA; 3 Department of Pediatrics, Keck School of Medicine, University of Southern California, Los Angeles, CA 90033, USA

In the original publication [1], there was a mistake in Table 2 when published. The mean and standard deviation column values for the following rows, Alexithymia—Identification, Alexithymia—Communication and Alexithymia—2 factor score were inaccurate. The corrected Table 2 appears below. The authors state that the scientific conclusions are unaffected. This correction was approved by the Academic Editor. The original publication has also been updated.

## Figures and Tables

**Table 2:** Group differences in empathy, alexithymia, affect recognition, and theory of mind.

	TD	ASD	Controlling for Age, Sex and FSIQ-4		Controlling for Age, Sex, FSIQ-4 and Alexithymia	
	Mean (±SD)	Mean (±SD)	*F*	*p*	*F*	*p*
IRI personal distress TD, n = 54; ASD, n = 56	12.65 (±5.27)	14.41 (±4.33)	5.020 *	0.027	0.781	0.379
IRI empathetic concern TD, n = 54; ASD, n = 56	18.37 (±4.84)	17.16 (±5.52)	0.396	0.530	0.002	0.967
IRI perspective taking TD, n = 54; ASD, n = 56	14.80 (±5.27)	12.59 (±6.01)	4.616 *	0.034	2.126	0.148
IRI fantasy TD, n = 54; ASD, n = 56	17.44 (±5.44)	16.73 (±5.72)	0.010	0.921	0.004	0.948
Alexithymia—Identification TD, n = 54; ASD, n = 56	0.48 (±0.40)	0.66 (±0.40)	3.058	0.083	-	-
Alexithymia—Communication TD, n = 54; ASD, n = 56	0.67 (±0.50)	1 (±0.45)	16.539 *	<0.001	-	-
Alexithymia—2 factor score TD, n = 54; ASD, n = 56	6.72 (±4.80)	9.59 (±4.32)	10.064 *	0.002	-	-
Affect recognition TD, n = 54; ASD, n = 56	11.80 (±2.34)	9.57 (±2.20)	10.767 *	0.001	6.163 *	0.015
Theory of Mind (Total) TD, n = 54; ASD, n = 54	25.39 (±2.00)	4.96 (±0.97)	4080.49 *	<0.001	3716.17 *	<0.001

ANCOVA was used to analyze differences between the TD and ASD groups. The test statistics found to be significant at a 95% confidence level are marked using *. TD = typically developing, ASD = autism spectrum disorder, FSIQ-4 = full-scale IQ, IRI = interpersonal reactivity index.
