# Supplementary material for: Relationships between Affect Recognition, Empathy, Alexithymia, and Co-Occurring Conditions in Autism
Source: Brain Sci. 2023 Aug 3;13(8):1161. doi: 10.3390/brainsci13081161 (PMC10452701; doi:10.3390/brainsci13081161)
Supplement: Supplementary file 1 [file brainsci-13-01161-s001.zip › brainsci-2545353-supplementary.pdf]

Supplementary

In addition to the analyses described in the main manuscript, we additionally looked at group differences in life satisfaction, as measured by the ladder (parent and child) and the life satisfaction scale, as well as relationships between life satisfaction and affect recognition and empathy. Although the autism spectrum disorder (ASD) group showed lower mean life satisfaction ($p<.001$), the between-group comparisons were not significant according to the ladder parent and child report ($t=2.005$, $p=.160$, and $t=3.328$, $p=.075$, Bonferroni corrected, respectively) and the life satisfaction ($t=3.074$, $p=.085$, Bonferroni corrected) scale. Further, there was no significant correlation between life satisfaction and affect recognition or empathy, or with other measures within or across groups.

Theory of mind (ToM), described as the cognitive ability to infer another's mental state, plays an important role in socialization [1] and autistic individuals are known to have an impairment in theory-of-mind capacity [2, 3]. Given the prior results with perspective taking, we were also interested in looking at how ToM, which largely involves perspective taking, is modulated and impacted across groups. Indeed, ToM difficulties are commonly found in ASD [4, 5]; although, this has recently been under scrutiny [6, 7]. In the current study, we replicate prior studies finding significant differences in ToM scores, measured using the NEPSY, between the TD and ASD groups [4, 5]. The differences remain significant, even when controlling for alexithymia. However, when evaluated using hierarchical linear regressions, ToM was not found to be a significant contributor to differences in perspective taking (cognitive empathy) across groups. This suggests that while both ToM and perspective taking enable understanding another person's perspective and facilitating an appropriate social response, they may not be modulated by similar factors. This is supported by prior research, which has found that while similar regions are involved in both processes, they have different underlying neuronal networks [8]. Further investigation into the interaction of these concepts and the generalizability of interventions is warranted.

Table S1: Factors correlated with affect recognition.

	TD (n=54)		ASD (n=56)	
	r	p	r	p
Age	-.116	.404	-.111	.416
VCI	.299	.028	.290	.030
PRI	.204	.139	.354*	.007
FSIQ-4	.291	.033	.377*	.004

Pearson's correlation was used to analyze the table and multiple comparisons correction was conducted using Bonferroni's method. The significant correlation coefficients at a 95% confidence level are marked using *. TD= Typically Developing, ASD = Autism Spectrum Disorder, FSIQ-4 = Full-Scale IQ, PRI = Perceptual Reasoning Index, VCI = Verbal Comprehension Index.

Table S2: Correlations between affect recognition and empathy and affect recognition and alexithymia in TD individuals.

	**Not controlled**		**Controlled for age and sex**		**Controlled for age, sex, and FSIQ-4**		**Controlled for age, sex, and PRI**		**Controlled for age, sex, and VCI**		**Controlled for age, sex, alexithymia, and FSIQ-4**	
	r	***p***	***r***	***p***	***r***	***p***	***r***	***p***	***r***	***p***	***r***	***p***

Alexithymia (Identification)	-.167	.228	-.208	.139	-.299	.033	-.266	.059	-.311*	.026	-	-
Alexithymia (Communication)	-.165	.234	-.226	.107	-.302	.031	-.269	.057	-.315*	.024	-	-
Alexithymia (2 factor)	-.181	.190	-.237	.090	-.332*	.017	-.294	.036	-.346*	.013	-	-
IRI empathetic concern	.082	.557	.099	.483	.077	.591	.089	.534	.068	.636	.062	.667
IRI personal distress	.080	.567	-.006	.966	-.087	.542	-.079	.579	-.057	.692	.078	.588
IRI perspective taking	-.133	.336	-.063	.659	-.053	.713	-.027	.852	-.093	.514	-.068	.640
IRI fantasy	.029	.833	.033	.816	-.050	.729	-.015	.914	-.045	.755	-.013	.928

Pearson's correlation was used to analyze the table and multiple comparisons correction was conducted using Bonferroni's method. The significant correlation coefficients at a 95% confidence level are marked using *. TD= Typically Developing, FSIQ-4 = Full-Scale IQ, PRI = Perceptual Reasoning Index, VCI = Verbal Comprehension Index, IRI = Interpersonal Reactivity Index.

Table S3 (a): Correlations between affect recognition and other ASD symptomology in the ASD and TD groups.

	TD		ASD	
	Controlled for age, sex, and FSIQ-4		Controlled for age, sex, and FSIQ-4	
	r	*p*	*r*	*p*
RBS Stereotype subscore TD, n=53; ASD, n=56	.141	.338	.095	.508
RBS Self-injury subscore TD, n=52; ASD, n=56	.116	.431	.163	.254
RBS Compulsive subscore TD, n=53; ASD, n=55	.221	.130	-.076	.597
RBS Ritual subscore TD, n=53; ASD, n=56	.320	.027	.019	.893
RBS Sameness subscore TD, n=53; ASD, n=55	.107	.469	.003	.984
RBS Restricted subscore TD, n=53; ASD, n=56	-.125	.395	.109	.448
CBCL Competence Activities T score	.051	.830	.197	.185

TD, n=53; ASD, n=54				
CBCL Competence Social T score TD, n=53; ASD, n=53	.277	.237	.028	.853
CBCL Competence School T score TD, n=53; ASD, n=54	-.100	.673	-.017	.908
CBCL Total score TD, n=53; ASD, n=53	.206	.385	.101	.498
CBCL Internalizing problems TD, n=49; ASD, n=51	-.034	.888	-.121	.419
CBCL Externalizing problems TD, n=49; ASD, n=52	-.449*	.047	-.107	.473
ADOS Social Affect ASD, n=55	-	-	-.255	.068
ADOS Restricted and Repetitive Behavior ASD, n=55	-	-	-.143	.311
ADOS Overall Total ASD, n=55	-	-	-.285	.040
ADIR Qualitative abnormalities in Reciprocal social interactions ASD, n=31	-	-	-.305	.114
ADIR Qualitative abnormalities in Communication ASD, n=31	-	-	.004	.982
ADIR Restricted Repetitive and Stereotyped patterns of behavior ASD, n=31	-	-	-.113	.567
SRS Restricted Interests and Repetitive Behavior TD, n=54; ASD, n=56	.221	.490	-.133	.499
SRS Social Communication and Interaction TD, n=54; ASD, n=56	-.113	.728	-.047	.810
SRS Total score TD, n=54; ASD, n=56	-.099	.760	.018	.926

Pearson's partial correlations were used to analyze the table and multiple comparisons correction was conducted using Bonferroni's method. The significant correlation coefficients at a 95% confidence level are marked using *. ASD = Autism Spectrum Disorder, FSIQ-4 = Full-Scale IQ; RBS = Repetitive Behavior Scale, SCARED = Screen for Child Anxiety Related Disorders, CBCL = Child Behavior Checklist, ASWS = Adolescent Sleep–Wake Scale, GSRS = Gastrointestinal Symptom Rating Scale, ADIR = Autism Diagnostic Interview-Revised, SRS = Social Responsiveness Scale.

Table S3 (b): Correlations between affect recognition and common conditions in ASD in the TD and ASD groups.

	TD		ASD	
	Controlled for age, sex, and FSIQ-4		Controlled for age, sex, and FSIQ-4	
	r	*p*	*r*	*p*
SCARED (Parent) Total score<br>TD, n=53; ASD, n=54	.092	.701	.081	.574
SCARED (Child) Total score<br>TD, n=49; ASD, n=54	-.195	.411	-.055	.704
ASWS Total score<br>TD, n=27; ASD, n=30	-.049	.837	.474*	.012
GSRS Total score<br>TD, n=41; ASD, n=43	.219	.354	.251	.226

Pearson’s partial correlations were used to analyze the table and multiple comparisons correction was conducted using Bonferroni’s method. The significant correlation coefficients at a 95% confidence level are marked using *. ASD = Autism Spectrum Disorder, FSIQ-4 = Full-Scale IQ; RBS = Repetitive Behavior Scale, SCARED = Screen for Child Anxiety Related Disorders, CBCL = Child Behavior Checklist, ASWS = Adolescent Sleep–Wake Scale, GSRS = Gastrointestinal Symptom Rating Scale, SRS = Social Responsiveness Scale.

Table S4: Correlation of personal distress with age and IQ.

	TD		ASD	
	r	*p*	*r*	*p*
**Age**	-.140	.314	.153	.259
**VCI**	.168	.225	-.163	.230
**PRI**	.185	.179	-.204	.131
**FSIQ-4**	.182	.187	-.206	.128

Pearson's correlation was used to analyze the table and multiple comparisons correction was conducted using Bonferroni’s method. VCI = Verbal Comprehension Index, PRI = Perceptual Reasoning Index, FSIQ-4 = Full-Scale IQ.

Table S5 (a) : Correlations between personal distress and other ASD symptomology in the ASD and TD groups.

	TD		ASD	
	Controlled for age, sex, and FSIQ-4		Controlled for age, sex, and FSIQ-4	

	r	***p***	***r***	***p***
RBS Stereotype subscore<br>TD, n=53; ASD, n=56	.250	.086	-.031	.827
RBS Self-injury subscore<br>TD, n=52; ASD, n=56	.120	.416	.120	.403
RBS Compulsive subscore<br>TD, n=53; ASD, n=55	.086	.562	.138	.336
RBS Ritual subscore<br>TD, n=53; ASD, n=56	.035	.811	.065	.651
RBS Sameness subscore<br>TD, n=53; ASD, n=55	.231	.114	-.010	.944
RBS Restricted subscore<br>TD, n=53; ASD, n=56	-.201	.171	.041	.778
CBCL Competence Activities T score<br>TD, n=53; ASD, n=54	-.064	.673	.011	.939
CBCL Competence Social T score<br>TD, n=53; ASD, n=53	.022	.887	.064	.668
CBCL Competence School T score<br>TD, n=53; ASD, n=54	-.258	.084	.287	.051
CBCL Total score<br>TD, n=53; ASD, n=53	-.076	.615	.162	.276
CBCL Internalizing problems<br>TD, n=49; ASD, n=51	.276	.063	.295	.044
CBCL Externalizing problems<br>TD, n=49; ASD, n=52	-.035	.819	-.189	.204
ADOS Social Affect<br>ASD, n=55	-	-	.147	.297
ADOS Restricted and Repetitive Behavior<br>ASD, n=55	-	-	-.167	.236
ADOS Overall Total<br>ASD, n=55	-	-	.074	.602
ADIR Qualitative abnormalities in Reciprocal social interactions<br>ASD, n=31	-	-	.017	.930
ADIR Qualitative abnormalities in Communication	-	-	.001	.996

ASD, n=31				
ADIR Restricted Repetitive and Stereotyped patterns of behavior<br>ASD, n=31	-	-	.147	.456
SRS Restricted Interests and Repetitive Behavior<br>TD, n=54; ASD, n=56	.118	.409	.077	.582
SRS Social Communication and Interaction<br>TD, n=54; ASD, n=56	-.040	.782	-.063	.656
SRS Total score<br>TD, n=54; ASD, n=56	-.018	.901	-.020	.889

Pearson's partial correlation was used to analyze the table and multiple comparisons correction was conducted using Bonferroni's method. The significant correlation coefficients at a 95% confidence level are marked using *. TD = Typically Developing, ASD = Autism Spectrum Disorder, FSIQ-4 = Full-Scale IQ, RBS = Repetitive Behavior Scale, SCARED = Screen for Child Anxiety Related Emotional Disorders, CBCL = Child Behavior Checklist, ASWS = Adolescent Sleep–Wake Scale, GSRS = Gastrointestinal Symptom Rating Scale, ADIR = Autism Diagnostic Interview-Revised, SRS = Social Responsiveness Scale.

Table S5 (b): Correlations between personal distress and common conditions in ASD in the TD and ASD groups.

	**TD**		**ASD**	
	**Controlled for age, sex, and FSIQ-4**		**Controlled for age, sex, and FSIQ-4**	
	r	***p***	***r***	***p***
SCARED (Parent) Total score<br>TD, n=53; ASD, n=54	.170	.238	.247	.081
SCARED (Child) Total score<br>TD, n=49; ASD, n=54	.574**	<.001	.581*	<.001
ASWS Total score<br>TD, n=27; ASD, n=30	-.014	.950	-.479*	.011
GSRS Total score<br>TD, n=41; ASD, n=43	.107	.524	.181	.264

Pearson's partial correlation was used to analyze the table and multiple comparisons correction was conducted using Bonferroni's method. The significant correlation coefficients at a 95% confidence level are marked using *. TD = Typically Developing, ASD = Autism Spectrum Disorder, FSIQ-4 = Full-Scale IQ, RBS = Repetitive Behavior Scale, SCARED = Screen for Child Anxiety Related Emotional Disorders, CBCL = Child Behavior Checklist, ASWS = Adolescent Sleep–Wake Scale, GSRS = Gastrointestinal Symptom Rating Scale, SRS = Social Responsiveness Scale.

Table S6: Correlation of empathetic concern with age and IQ.

	TD		ASD	
	r	*p*	*r*	*p*
Age	-.027	.844	.157	.248
VCI	.118	.395	.100	.461
PRI	.048	.732	.288	.032
FSIQ-4	.083	.551	.211	.119

Pearson's partial correlation was used to analyze the table and multiple comparisons correction was conducted using Bonferroni's method. TD = Typically Developing, ASD = Autism Spectrum Disorder, FSIQ-4 = Full-Scale IQ, PRI = Perceptual Reasoning Index, VCI = Verbal Comprehension Index.

Table S7 (a) : Correlations between empathetic concern and other ASD symptomology in the ASD and TD groups.

	TD		ASD	
	**Controlled for age, sex, and FSIQ-4**		**Controlled for age, sex, and FSIQ-4**	
	r	*p*	*r*	*p*
RBS Stereotype subscore<br>TD, n=53; ASD, n=56	.119	.447	-.084	.774
RBS Self-injury subscore<br>TD, n=52; ASD, n=56	.314	.040	-.147	.617
RBS Compulsive subscore<br>TD, n=53; ASD, n=55	.191	.220	.127	.666
RBS Ritual subscore<br>TD, n=53; ASD, n=56	.063	.688	.094	.750
RBS Sameness subscore<br>TD, n=53; ASD, n=55	.220	.156	.228	.433
RBS Restricted subscore<br>TD, n=53; ASD, n=56	.071	.650	-.053	.857
CBCL Competence Activities T score<br>TD, n=53; ASD, n=54	-.189	.811	-.254	.382
CBCL Competence Social T score<br>TD, n=53; ASD, n=53	.090	.910	.320	.265

CBCL Competence School T score<br>TD, n=53; ASD, n=54	.436	.564	-.213	.465
CBCL Total score<br>TD, n=53; ASD, n=53	.006	.994	.067	.820
CBCL Internalizing problems<br>TD, n=49; ASD, n=51	-.242	.758	-.164	.575
CBCL Externalizing problems<br>TD, n=49; ASD, n=52	-.225	.775	-.227	.435
ADOS Social Affect<br>ASD, n=55	-	-	-.078	.585
ADOS Restricted and Repetitive Behavior<br>ASD, n=55	-	-	-.150	.290
ADOS Overall Total<br>ASD, n=55	-	-	-.125	.376
ADIR Qualitative abnormalities in Reciprocal social interactions<br>ASD, n=31	-	-	.178	.543
ADIR Qualitative abnormalities in Communication<br>ASD, n=31	-	-	.222	.447
ADIR Restricted Repetitive and Stereotyped patterns of behavior<br>ASD, n=31	-	-	-.162	.580
SRS Restricted Interests and Repetitive Behavior<br>TD, n=54; ASD, n=56	-.415	.585	.099	.735
SRS Social Communication and Interaction<br>TD, n=54; ASD, n=56	-.710	.290	-.107	.716
SRS Total score<br>TD, n=54; ASD, n=56	-.637	.363	-.037	.899

Pearson's partial correlation was used to analyze the table and multiple comparisons correction was conducted using Bonferroni’s method. The significant correlation coefficients at a 95% confidence level are marked using *. TD = Typically Developing, ASD = Autism Spectrum Disorder, FSIQ-4 = Full-Scale IQ, RBS = Repetitive Behavior Scale, SCARED = Screen for Child Anxiety Related Emotional Disorders, CBCL = Child Behavior Checklist, ASWS = Adolescent Sleep–Wake Scale, GSRS = Gastrointestinal Symptom Rating Scale.

Table S7 (b): Correlations between empathetic concern and common conditions in ASD in the TD and ASD groups.

	TD		ASD	
	Controlled for age, sex, and FSIQ-4		Controlled for age, sex, and FSIQ-4	
	r	*p*	*r*	*p*
SCARED (Parent) Total score<br>TD, n=53; ASD, n=54	.125	.424	-.331	.248
SCARED (Child) Total score<br>TD, n=49; ASD, n=54	.168	.280	.039	.894
ASWS Total score<br>TD, n=27; ASD, n=30	.011	.989	.207	.478
GSRS Total score<br>TD, n=41; ASD, n=43	-.729	.271	.199	.496

Pearson's partial correlation was used to analyze the table and multiple comparisons correction was conducted using Bonferroni's method. The significant correlation coefficients at a 95% confidence level are marked using *. TD = Typically Developing, ASD = Autism Spectrum Disorder, FSIQ-4 = Full-Scale IQ, RBS = Repetitive Behavior Scale, SCARED = Screen for Child Anxiety Related Emotional Disorders, CBCL = Child Behavior Checklist, ASWS = Adolescent Sleep–Wake Scale, GSRS = Gastrointestinal Symptom Rating Scale.

Table S8: Correlation of perspective taking with age and IQ.

	TD (n = 54)		ASD (n = 56)	
	r	*p*	*r*	*p*
Age	.215	.118	.326*	.014
VCI	.063	.649	.192	.156
PRI	-.065	.639	.153	.261
FSIQ-4	-.005	.971	.207	.125

Pearson's partial correlation was used to analyze the table. The significant correlation coefficients at a 95% confidence level are marked using *. TD = Typically Developing, ASD = Autism Spectrum Disorder, FSIQ-4 = Full-Scale IQ, PRI = Perceptual Reasoning Index, VCI = Verbal Comprehension Index.

Table S9 (a) : Correlations between perspective taking and other ASD symptomology in the ASD and TD groups.

	TD	ASD
	Controlled for age, sex, and FSIQ-4	Controlled for age, sex, and FSIQ-4

	r	*p*	*r*	*p*
Theory of Mind<br>TD, n=54; ASD, n=54	-.091	.524	-.207	.146
RBS Stereotype subscore<br>TD, n=53; ASD, n=56	-.162	.272	-.227	.102
RBS Self-injury subscore<br>TD, n=52; ASD, n=56	.177	.228	-.295	.036
RBS Compulsive subscore<br>TD, n=53; ASD, n=55	.133	.367	-.309	.028
RBS Ritual subscore<br>TD, n=53; ASD, n=56	-.150	.307	-.322	.021
RBS Sameness subscore<br>TD, n=53; ASD, n=55	-.031	.835	-.246	.082
RBS Restricted subscore<br>TD, n=53; ASD, n=56	.052	.723	-.181	.204
CBCL Competence Activities T score<br>TD, n=53; ASD, n=54	.139	.356	-.010	.945
CBCL Competence Social T score<br>TD, n=53; ASD, n=53	.259	.082	.074	.622
CBCL Competence School T score<br>TD, n=53; ASD, n=54	.272	.067	.176	.237
CBCL Total score<br>TD, n=53; ASD, n=53	.332*	.024	.047	.754
CBCL Internalizing problems<br>TD, n=49; ASD, n=51	.025	.867	-.280	.056
CBCL Externalizing problems<br>TD, n=49; ASD, n=52	-.160	.288	-.286	.051
ADOS Social Affect<br>ASD, n=55	-	-	-.016	.909
ADOS Restricted and Repetitive Behavior<br>ASD, n=55	-	-	.138	.330
ADOS Overall Total<br>ASD, n=55	-	-	.035	.805
ADIR Qualitative abnormalities in Reciprocal social interactions<br>ASD, n=31	-	-	.253	.067

ADIR Qualitative abnormalities in Communication<br>ASD, n=31	-	-	.271	.171
ADIR Restricted Repetitive and Stereotyped patterns of behavior<br>ASD, n=31	-	-	-.256	.111
SRS Restricted Interests and Repetitive Behavior<br>TD, n=54; ASD, n=56	.059	.680	.256	.189
SRS Social Communication and Interaction<br>TD, n=54; ASD, n=56	.171	.230	.141	.475
SRS Total score<br>TD, n=54; ASD, n=56	.183	.198	-.010	.959

Pearson's partial correlation was used to analyze the table and multiple comparisons correction was conducted using Bonferroni's method. The significant correlation coefficients at a 95% confidence level are marked using *. TD = Typically Developing, ASD = Autism Spectrum Disorder, FSIQ-4 = Full-Scale IQ, RBS = Repetitive Behavior Scale, SCARED = Screen for Child Anxiety Related Emotional Disorders, CBCL = Child Behavior Checklist, ASWS = Adolescent Sleep–Wake Scale, GSRS = Gastrointestinal Symptom Rating Scale.

Table S9 (b): Correlations between perspective taking and common conditions in ASD in the TD and ASD groups.

	**TD**		**ASD**	
	**Controlled for age, sex, and FSIQ-4**		**Controlled for age, sex, and FSIQ-4**	
	r	***p***	***r***	***p***
SCARED (Parent) Total score<br>TD, n=53; ASD, n=54	-.078	.589	-.322	.021
SCARED (Child) Total score<br>TD, n=49; ASD, n=54	-.006	.969	-.246	.082
ASWS Total score<br>TD, n=27; ASD, n=30	.102	.635	-.072	.614
GSRS Total score<br>TD, n=41; ASD, n=43	-.194	.244	-.063	.652

Pearson's partial correlation was used to analyze the table and multiple comparisons correction was conducted using Bonferroni's method. TD = Typically Developing, ASD = Autism Spectrum Disorder, FSIQ-4 = Full-Scale IQ, RBS = Repetitive Behavior Scale, SCARED = Screen for Child Anxiety Related Emotional Disorders, CBCL = Child Behavior Checklist, ASWS = Adolescent Sleep–Wake Scale, GSRS = Gastrointestinal Symptom.
Rating Scale

Table S10: Correlation of fantasy with age and IQ.

	TD		ASD	
	r	*p*	*r*	*p*
Age	-.018	.897	.127	.350
VCI	.251	.067	.053	.698
PRI	.172	.214	.034	.804
FSIQ-4	.237	.085	.046	.734

Spearman's correlation was used to analyze the table and multiple comparisons correction was conducted using Bonferroni's method. The significant correlation coefficients at a 95% confidence level are marked using *. FSIQ-4 = Full-Scale IQ, PRI = Perceptual Reasoning Index, VCI = Verbal Comprehension Index.

Table S11(a): Correlations between fantasy and other ASD symptomology in the ASD and TD groups.

	TD		ASD	
	**Controlled for age, sex, and FSIQ-4**		**Controlled for age, sex, and FSIQ-4**	
	r	*p*	*r*	*p*
Theory of Mind<br>TD, n=54; ASD, n=54	-.138	.334	-.241	.089
RBS Stereotype subscore<br>TD, n=53; ASD, n=56	.070	.637	.233	.423
RBS Self-injury subscore<br>TD, n=52; ASD, n=56	.280	.054	-.043	.885
RBS Compulsive subscore<br>TD, n=53; ASD, n=55	.158	.284	.276	.339
RBS Ritual subscore<br>TD, n=53; ASD, n=56	.005	.973	.089	.762
RBS Sameness subscore<br>TD, n=53; ASD, n=55	.197	.180	.114	.698
RBS Restricted subscore<br>TD, n=53; ASD, n=56	.116	.433	.165	.572
CBCL Competence Activities T score<br>TD, n=53; ASD, n=54	.012	.938	.069	.816

CBCL Competence Social T score<br>TD, n=53; ASD, n=53	.221	.140	-.112	.702
CBCL Competence School T score<br>TD, n=53; ASD, n=54	.105	.486	-.184	.528
CBCL Total score<br>TD, n=53; ASD, n=53	.200	.182	-.120	.682
CBCL Internalizing problems<br>TD, n=49; ASD, n=51	-.017	.911	-.209	.474
CBCL Externalizing problems<br>TD, n=49; ASD, n=52	.045	.767	-.196	.502
ADOS Social Affect<br>ASD, n=55	-	-	.002	.991
ADOS Restricted and Repetitive Behavior<br>ASD, n=55	-	-	.114	.421
ADOS Overall Total<br>ASD, n=55	-	-	.043	.763
ADIR Qualitative abnormalities in Reciprocal social interactions<br>ASD, n=31	-	-	.264	.361
ADIR Qualitative abnormalities in Communication<br>ASD, n=31	-	-	.037	.899
ADIR Restricted Repetitive and Stereotyped patterns of behavior<br>ASD, n=31	-	-	-.356	.211
SRS Restricted Interests and Repetitive Behavior<br>TD, n=54; ASD, n=56	.149	.297	.221	.448
SRS Social Communication and Interaction<br>TD, n=54; ASD, n=56	.217	.126	-.127	.666
SRS Total score<br>TD, n=54; ASD, n=56	.248	.079	-.130	.354

Pearson's partial correlation was used to analyze the table and multiple comparisons correction was conducted using Bonferroni’s method. The significant correlation coefficients at a 95% confidence level are marked using *. TD = Typically Developing, ASD = Autism Spectrum Disorder, FSIQ-4 = Full-Scale IQ, RBS = Repetitive Behavior Scale, SCARED = Screen for Child Anxiety Related Emotional Disorders, CBCL = Child Behavior Checklist, ASWS = Adolescent Sleep–Wake Scale, GSRS = Gastrointestinal Symptom Rating Scale, ADIR = Autism Diagnostic Interview-Revised.

Table S11(b): Correlations between fantasy and common conditions in ASD in the TD and ASD groups.

	TD		ASD	
	Controlled for age, sex, and FSIQ-4		Controlled for age, sex, and FSIQ-4	
	r	p	r	p
SCARED (Parent) Total score<br>TD, n=53; ASD, n=54	-.121	.403	-.304	.291
SCARED (Child) Total score<br>TD, n=49; ASD, n=54	.027	.859	-.086	.770
ASWS Total score<br>TD, n=27; ASD, n=30	.105	.625	.161	.582
GSRS Total score<br>TD, n=41; ASD, n=43	-.153	.360	.030	.919

Pearson's partial correlation was used to analyze the table and multiple comparisons correction was conducted using Bonferroni's method. The significant correlation coefficients at a 95% confidence level are marked using *. TD = Typically Developing, ASD = Autism Spectrum Disorder, FSIQ-4 = Full-Scale IQ, RBS = Repetitive Behavior Scale, SCARED = Screen for Child Anxiety Related Emotional Disorders, CBCL = Child Behavior Checklist, ASWS = Adolescent Sleep–Wake Scale, GSRS = Gastrointestinal Symptom Rating Scale, ADIR = Autism Diagnostic Interview-Revised.

Table S12 (a): Correlations between alexithymia and empathy in the TD group.

	Alexithymia (Identification)		Alexithymia (Communication)		Alexithymia - 2 factor	
	Controlled for age, sex, and FSIQ-4		Controlled for age, sex, and FSIQ-4		Controlled for age, sex, and FSIQ-4	
	r	*p*	*r*	*p*	*r*	*p*
IRI personal distress	.429**	.002	.405**	.003	.461*	<.001
IRI empathic concern	.069	.631	-.184	.196	-.056	.698
IRI perspective taking	.077	.592	-.151	.292	-.034	.814
IRI fantasy	.128	.369	.072	.617	.113	.431

Pearson's partial correlation was used to analyze the table and multiple comparisons correction was conducted using Bonferroni's method. The significant correlation coefficients at a 95% confidence level are marked using *. TD = Typically Developing, ASD = Autism Spectrum Disorder, FSIQ-4 = Full-Scale IQ.

Table S12 (b): Correlations between alexithymia and empathy in the ASD group.

	**Alexithymia (Identification)**		**Alexithymia (Communication)**		**Alexithymia - 2 factor**	
	**Controlled for age, sex, and FSIQ-4**		**Controlled for age, sex, and FSIQ-4**		**Controlled for age, sex, and FSIQ-4**	
	r	***p***	***r***	***p***	***r***	***p***
IRI personal distress	.380*	.005	.309*	.024	.401*	.003
IRI empathic concern	.002	.986	-.523*	<.001	-.276	.046
IRI perspective taking	-.227	.102	-.340	.013	-.321	.019
IRI fantasy	-.103	.464	-.346	.011	-.247	.075

Pearson's partial correlation was used to analyze the table and multiple comparisons correction was conducted using Bonferroni's method. The significant correlation coefficients at a 95% confidence level are marked using *. TD = Typically Developing, ASD = Autism Spectrum Disorder, FSIQ-4 = Full-Scale IQ.

References

1. Schlaffke, L.; Lissek, S.; Lenz, M.; Juckel, G.; Schultz, T.; Tegenthoff, M.; Schmidt-Wilcke, T.; Brüne, M. Shared and nonshared neural networks of cognitive and affective theory-of-mind:A neuroimaging study using cartoon picture stories. *Human brain mapping* **2015**, *36* (1), 29-39.DOI: 10.1002/hbm.22610.
2. Baron-Cohen, S. Mindblindness: An essay on autism and theory of mind; The MIT Press, 1995.
3. Begeer, S.; Gevers, C.; Clifford, P.; Verhoeve, M.; Kat, K.; Hoddenbach, E.; Boer, F. Theory ofMind training in children with autism: a randomized controlled trial. *Journal of autism and developmental disorders* **2011**, *41* (8), 997-1006. DOI: 10.1007/s10803-010-1121-9.
4. Baron-Cohen, S.; Ring, H. A.; Bullmore, E. T.; Wheelwright, S.; Ashwin, C.; Williams, S. C. R.The amygdala theory of autism. *Neuroscience and biobehavioral reviews* **2000**, *24* (3), 355-364. DOI: 10.1016/S0149-7634(00)00011-7.
5. Happé, F.; Frith, U. Theory of mind in autism. In *Learning and cognition in autism.*, Current issues in autism., Plenum Press, 1995; pp 177-197.
6. Livingston, L. A.; Carr, B.; Shah, P. Recent Advances and New Directions in Measuring Theory of Mind in Autistic Adults. *Journal of autism and developmental disorders* **2019**, *49* (4), 1738-1744. DOI: 10.1007/s10803-018-3823-3.
7. Pedreño, C.; Pousa, E.; Navarro, J. B.; Pàmias, M.; Obiols, J. E. Exploring the Components ofAdvanced Theory of Mind in Autism Spectrum Disorder. *Journal of autism and developmentaldisorders* **2017**, *47* (8), 2401-2409. DOI: 10.1007/s10803-017-3156-7.
8. Preckel, K.; Kanske, P.; Singer, T. On the interaction of social affect and cognition: empathy, compassion and theory of mind. *Current opinion in behavioral sciences* **2018**, *19*, 1-6. DOI: 10.1016/j.cobeha.2017.07.010.
